# Milk-Derived Exosomes as Nanocarriers to Deliver Curcumin and Resveratrol in Breast Tissue and Enhance Their Anticancer Activity

**DOI:** 10.3390/ijms23052860

**Published:** 2022-03-05

**Authors:** Antonio González-Sarrías, Carlos E. Iglesias-Aguirre, Adrián Cortés-Martín, Fernando Vallejo, Alice Cattivelli, Lorena del Pozo-Acebo, Andrea Del Saz, María Carmen López de las Hazas, Alberto Dávalos, Juan Carlos Espín

**Affiliations:** 1 Laboratory of Food and Health, Research Group on Quality, Safety, and Bioactivity of Plant Foods, Department Food Science and Technology, CEBAS-CSIC, P.O. Box 164, Campus de Espinardo, 30100 Murcia, Spain; agsarrias@cebas.csic.es (A.G.-S.); ceiglesias@cebas.csic.es (C.E.I.-A.); acortesmartin@ucc.ie (A.C.-M.); fvallejo@cebas.csic.es (F.V.); alice.cattivelli@unimore.it (A.C.); 2 APC Microbiome Ireland & School of Microbiology, University College Cork, T12 YT20 Cork, Ireland; 3 Department of Life Sciences, University of Modena and Reggio Emilia, Via Amendola 2—Pad. Besta, 42100 Reggio Emilia, Italy; 4 Laboratory of Epigenetics of Lipid Metabolism, Madrid Institute for Advanced Studies (IMDEA)-Food, CEI UAM + CSIC, 28049 Madrid, Spain; lorena.delpozo@imdea.org (L.d.P.-A.); andrea.delsaz@imdea.org (A.D.S.); mcarmen.lopez@imdea.org (M.C.L.d.l.H.); alberto.davalos@imdea.org (A.D.)

**Keywords:** exosome, polyphenol, resveratrol, curcumin, breast cancer, apoptosis, metabolism, nanocarrier, ABC transporters

## Abstract

Dietary (poly)phenols are extensively metabolized, limiting their anticancer activity. Exosomes (EXOs) are extracellular vesicles that could protect polyphenols from metabolism. Our objective was to compare the delivery to breast tissue and anticancer activity in breast cancer cell lines of free curcumin (CUR) and resveratrol (RSV) vs. their encapsulation in milk-derived EXOs (EXO-CUR and EXO-RSV). A kinetic breast tissue disposition was performed in rats. CUR and RSV were analyzed using UPLC-QTOF-MS and GC-MS, respectively. Antiproliferative activity was tested in MCF-7 and MDA-MB-231 breast cancer and MCF-10A non-tumorigenic cells. Cell cycle distribution, apoptosis, caspases activation, and endocytosis pathways were determined. CUR and RSV peaked in the mammary tissue (41 ± 15 and 300 ± 80 nM, respectively) 6 min after intravenous administration of EXO-CUR and EXO-RSV, but not with equivalent free polyphenol concentrations. Nanomolar EXO-CUR or EXO-RSV concentrations, but not free CUR or RSV, exerted a potent antiproliferative effect on cancer cells with no effect on normal cells. Significant (*p* < 0.05) cell cycle alteration and pro-apoptotic activity (via the mitochondrial pathway) were observed. EXO-CUR and EXO-RSV entered the cells primarily via clathrin-mediated endocytosis, avoiding ATP-binding cassette transporters (ABC). Milk EXOs protected CUR and RSV from metabolism and delivered both polyphenols to the mammary tissue at concentrations compatible with the fast and potent anticancer effects exerted in model cells. Milk EXOs enhanced the bioavailability and anticancer activity of CUR and RSV by acting as Trojan horses that escape from cancer cells’ ABC-mediated chemoresistance.

## 1. Introduction

Exosomes (EXOs) are a subset of extracellular vesicles secreted by cells that regulate intercellular communication in organisms [1,2]. EXOs can transfer their cargo, consisting of different metabolites, lipids, functional proteins, and nucleic acids, and modulate the response of distant recipient cells [1,2,3,4,5]. EXOs can modulate tissue repair, immune response, cell maintenance, and trafficking in both normal and pathological processes, including neurodegeneration, inflammation, cancer, and cardiovascular diseases [6,7,8,9]. Moreover, EXOs can cross physical barriers, including the blood–brain barrier and placenta [10]. In this scenario, EXOs have emerged as molecule delivery vehicles, including for drugs, natural bioactives, and nucleic acids, with potential clinical applications [11,12,13,14]. 

An increasing number of studies show that food-derived EXOs may be relevant to the biological food effects and would therefore be of biotechnological interest [15,16]. Milk, especially, possesses advantages such as cross-species biocompatibility and inert toxicity, and the ability to cross the blood–brain barrier and act as a vehicle for both hydrophilic and lipophilic macromolecules. In addition, it is possible to develop technology to modify EXOs’ surfaces to ensure tissue-specific biodistribution [17,18]. In this regard, milk is a scalable source of EXOs, highly recommended for therapeutic applications [19,20]. Milk-derived EXOs have been previously used to encapsulate and deliver chemotherapy drugs, natural compounds, and microRNAs [13,21,22,23]. Overall, EXOs protect their cargo from gastrointestinal digestion, metabolism, and degradation, allowing its potential biodistribution in systemic tissues, including the brain [24,25,26]. 

Dietary (poly)phenols have been acknowledged to have a plethora of biological properties, including cancer chemopreventive activity [27]. This activity has been reported to be mediated in animal models by the decrease in K67, Bcl-2/Bax ratio, angiogenesis, matrix metalloproteinases, and cytokines, and an increase in p21, endostatin, and caspases activation, among many other mechanisms [28]. However, they are poorly bioavailable, i.e., the fraction of intact (poly)phenolics ingested that reach the bloodstream is low [27,29]. In addition, once absorbed, phase-II enzymes (mainly glucuronyl, sulfate, and catechol-methyl transferases) extensively metabolize (poly)phenols, yielding conjugated metabolites, primarily glucuronides and sulfates, that can show some activity but much less than their food-occurring phenolic precursors [29,30,31,32]. In contrast to animal models, the relatively low dietary polyphenol doses consumed along with phase-II metabolism preclude a strong polyphenol-related activity in humans. Therefore, preserving (poly)phenols such as curcumin (CUR) and resveratrol (RSV) from being metabolized is an attractive approach to enhance their anticancer bioactivity in systemic tissues [32]. Both CUR and RSV are polyphenols with acknowledged cancer chemopreventive properties and have been used as supplements to manage various inflammatory conditions [33,34]. However, they show poor bioavailability and hardly reach human systemic tissues as free, non-conjugated polyphenols [31,32].

In addition to metabolism, the ATP-binding cassette (ABC) transporters can hamper the anticancer activity of drugs and (poly)phenols [34,35]. These transporters can be overexpressed in cancer cells, including breast cancer cells, limiting the entry of free and conjugated (poly)phenols into the cells and (or) driving their efflux back into the extracellular space [34,36,37]. 

Considering the above, we hypothesize that encapsulating (poly)phenols such as CUR and RSV in milk-derived EXOs could avoid their intense cellular metabolism and restriction by ABC transporters. Therefore, we aimed to evaluate whether milk-derived EXOs that incorporate CUR or RSV (EXO-CUR and EXO-RSV, respectively) can reach the mammary tissue, bypassing their metabolism. Next, we will compare and characterize the antiproliferative activity of free vs. encapsulated polyphenols in human breast cancer and non-tumorigenic cells, using the observed in vivo breast-occurring CUR and RSV concentrations. 

## 2. Results

### 2.1. Encapsulation of CUR and RSV into Milk-Derived EXOs

**Figure 1:**
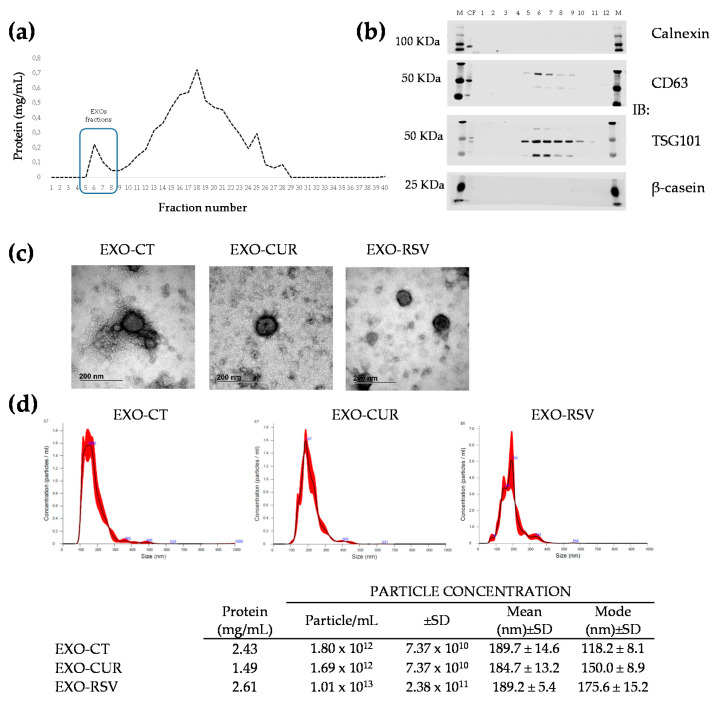
(**a**) Protein concentration (mg/mL) of isolated and purified EXOs in SEC fractions. (**b**) Western blot analysis of proteins present (CD63 and TSG101) or absent (calnexin and β-casein) in EXO fractions. (**c**) Transmission electron microscopy observations of milk-derived EXOs isolated via the passive incubation method (scale bar: 200 nm). (**d**) Size and distribution profiles of milk-derived EXOs (EXO-CT, EXO-CUR, and EXO-RSV) isolated via the passive incubation method.

We tested three different methods and conditions of CUR and RSV encapsulation into EXOs. EXOs were first isolated by ultracentrifugation (UC), loaded with polyphenols by either sonication, electroporation, or passive incubation, and further purified by size exclusion chromatography (SEC). Purified exosome fractions (F5 to F9) from SEC were combined for further analysis (Figure 1a). The proteins of milk-derived EXOs were verified by Western blot analysis (Figure 1b and Figure S1). Milk-derived EXOs contained the EXO marker proteins CD63 and TSG101, whereas other non-EXO proteins (calnexin and β-casein) were absent. Sonication and electroporation resulted in a limited RSV and CUR incorporation (<2 µM). In contrast, passive incubation yielded the highest CUR (2 μM) and RSV (15 μM) concentrations. Electron microscopy analysis showed similar sizes and shapes of EXOs, whether loaded or non-loaded (Figure 1c). Nanoparticle tracking analysis showed similar particle size and size distribution (Figure 1d). Overall, the data show that polyphenol loading did not change EXOs’ major characteristics. For downstream experiments, we used passive diffusion as the selected method. 

### 2.2. CUR and RSV Detection in Breast Tissue after Free and EXO-Encapsulated Polyphenols Administration

**Figure 2:**
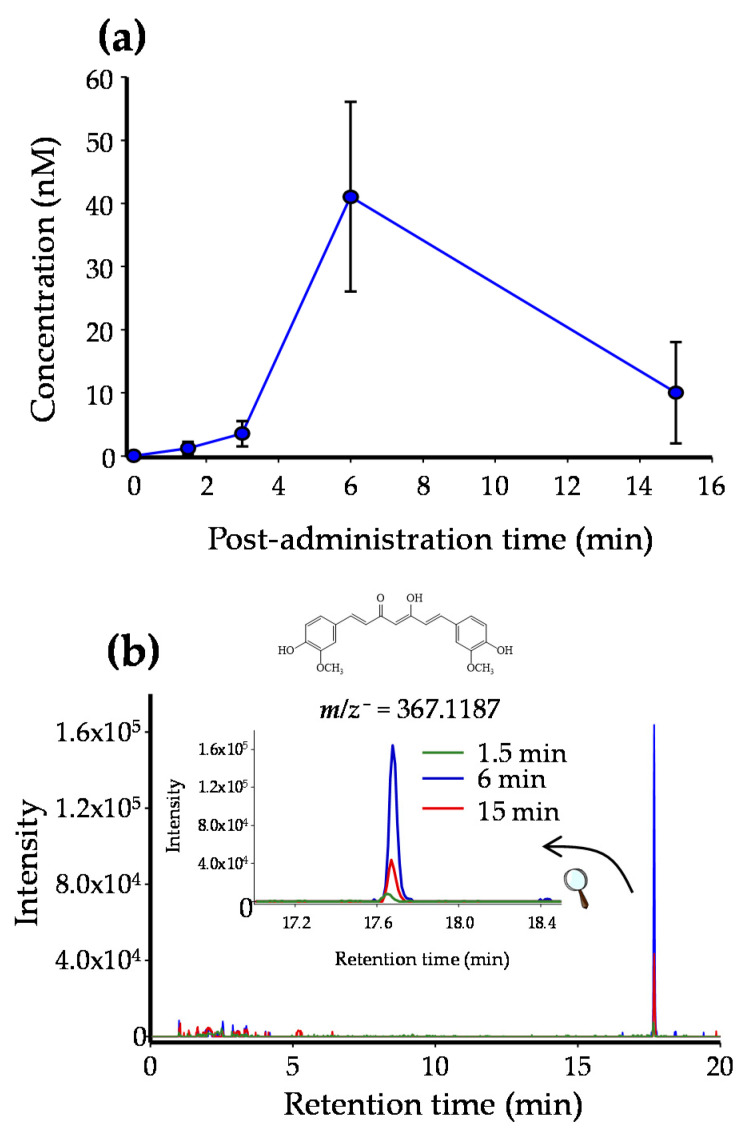
(**a**) CUR kinetic distribution in the rat mammary tissue. (**b**) Extracted ion chromatograms show CUR’s molecular ion intensities after 1.5, 6, and 15 min of EXO-CUR intravenous administration. A minimum of 3 animals were used for each time point. Data are shown as mean ± standard deviation (SD).

CUR and RSV (both free and encapsulated in milk-derived EXOs) were administered in female rats. Figure 2 shows the pharmacokinetic distribution of CUR in the rat mammary gland after administering EXO-CUR, with a peak of free CUR (41 ± 15 nM) in the tissue at 6 min post administration. In the case of EXO-RSV, free RSV also peaked (300 ± 80 nM) at 6 min post EXO-RSV administration and followed a similar kinetic profile as CUR (Figure S2). In both cases, traces (not quantified) of CUR-glucuronide and RSV-glucuronide were also detected. Neither CUR nor RSV was detected at any time of testing in the mammary tissue when non-encapsulated CUR and RSV were administered (results not shown).

### 2.3. Effect of CUR and RSV on the Viability of Cancerous and Normal Breast Cells (Free Polyphenols vs. Polyphenols Encapsulated into EXOs)

First, we calculated the maximum milk-derived EXO percentage in the cell culture without affecting the osmolarity of the cell medium. This percentage was 7.5%. Therefore, most of the experiments were carried out at 2.5% EXO (equivalent to 48 nM EXO-CUR and 375 nM EXO-RSV) or 5% EXO (equivalent to 96 nM EXO-CUR and 750 nM EXO-RSV).

**Figure 3:**
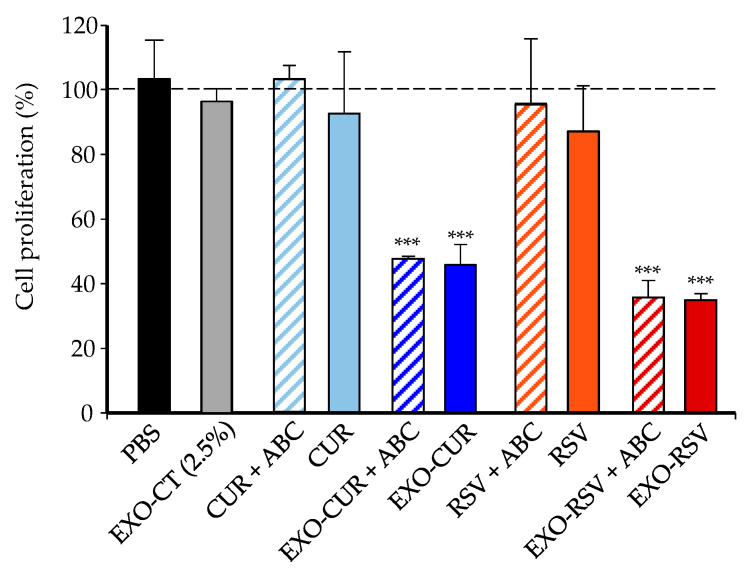
Cell proliferation of MCF-7 cells after treatment with free CUR (48 nM), free RSV (375 nM), EXO-CUR (48 nM), and EXO-RSV (375 nM), in the presence or absence of a mixture of ABC transporter inhibitors (1 µM each) after 72 h. *** *p* < 0.001 (EXO-CUR and EXO-RSV vs. EXO-CT). EXO-CT: milk control exosomes; ABC: transporter inhibitors CP100356 (P-glycoprotein (P-gp) inhibitor), Ko143 (breast cancer resistant protein (BCRP) inhibitor), and probenecid (multidrug-resistant protein (MRP) inhibitor). Data are presented as the mean ± SD of at least three independent experiments (n = 6 wells per experiment).

Next, we compared the possible role of ABC transporters in the antiproliferative activity of RSV and CUR vs. their encapsulated forms EXO-RSV and EXO-CUR. Figure 3 shows that free CUR (48 nM) and RSV (375 nM) did not exert significant antiproliferative activity in MCF-7 cells, and activity was even less in the presence of the ABC inhibitors. However, when encapsulated into milk-derived EXOs, the same polyphenol concentrations exerted a robust antiproliferative activity, which was not affected by the presence of ABC inhibitors (Figure 3).

**Figure 4:**
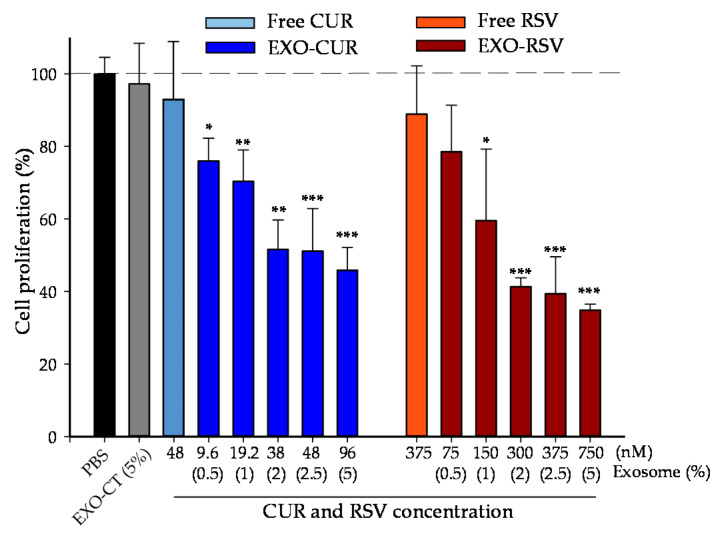
Comparison of the effect of free CUR and RSV vs. EXO-CUR and EXO-RSV on MCF-7 cell proliferation after 72 h. * *p* < 0.05; ** *p* < 0.01; *** *p* < 0.001 (EXO-CUR and EXO-RSV vs. EXO-CT). EXO-CT: non-loaded milk-derived EXOs. The X-axis shows the concentration of CUR and RSV that the EXOs incorporate (nM) and, below, the equivalent % of the fraction of EXOs tested in the cell media. Data are presented as the mean ± SD of at least three independent experiments (n = 6 wells per experiment).

**Figure 5:**
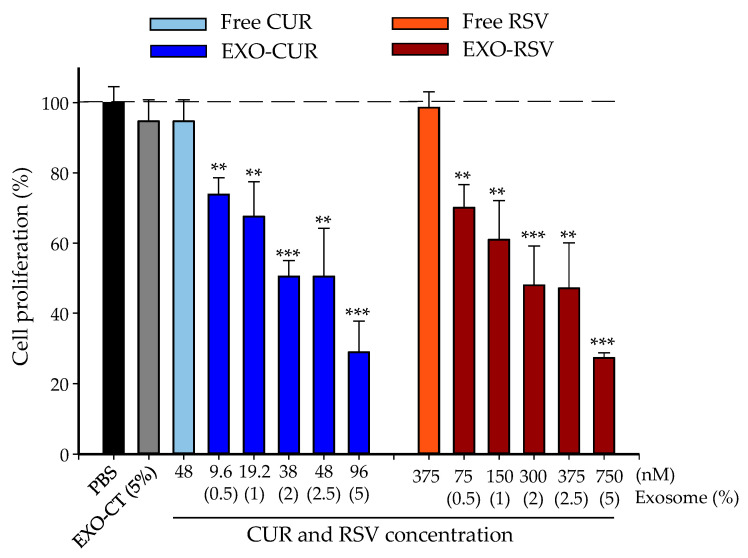
Comparison of the effect of free CUR and RSV vs. EXO-CUR and EXO-RSV on MDA-MB-231 cell proliferation after 72 h. The asterisks indicate statistically significant differences between EXO-CUR and EXO-RSV vs. control (non-loaded) EXOs (EXO-CT). ** *p* < 0.01; *** *p* < 0.001. The X-axis shows the concentration of CUR and RSV incorporated by EXOs and, below, the % of the EXO fraction tested in the cell media. Data are presented as the mean ± SD of at least three independent experiments (n = 6 wells per experiment).

Then, we evaluated the dose-dependent antiproliferative effect of free CUR and RSV vs. EXO-CUR and EXO-RSV in different human breast cell lines, i.e., MCF-7 (p53 wild breast cancer cells), MDA-MB-231 (p53 mutant breast cancer cells), and MCF-10A (non-tumorigenic). Figure 4 and Figure 5 show the antiproliferative activity in MCF-7 and MDA-MB-231, respectively, whereas no antiproliferative effect was detected with either free or EXO-encapsulated polyphenols in MCF-10A non-tumorigenic breast cells (Figure S3).

### 2.4. Effect on Cell Cycle Distribution and Apoptosis

**Figure 6:**
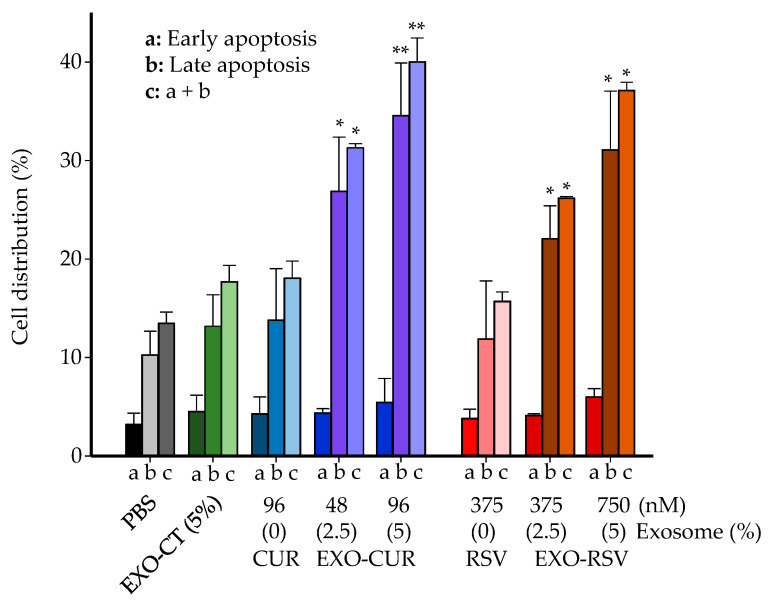
Comparison of the apoptosis induction exerted by CUR and RSV vs. EXO-CUR and EXO-RSV in MCF-7 after 72 h. EXO-CT: non-loaded milk-derived EXOs. * *p* < 0.05; ** *p* < 0.01 (significant differences for EXO-CUR and EXO-RSV vs. EXO-CT). Data are shown as the mean ± SD of 3 independent experiments (2 wells per treatment) for each time point.

Next, we delved into the mechanisms involved in the antiproliferative effect, focusing on the MCF-7 cell line. A significant increase in the G_0_/G_1_ phase and decrease in the S phase was observed after treatment with EXO-CUR and EXO-RSV, but not with free CUR or RSV (Figure S4). In addition, a dose-dependent apoptotic induction was observed when MCF-7 cells were treated with EXO-CUR or EXO-RSV but not with the corresponding free polyphenols (Figure 6).

**Figure 7:**
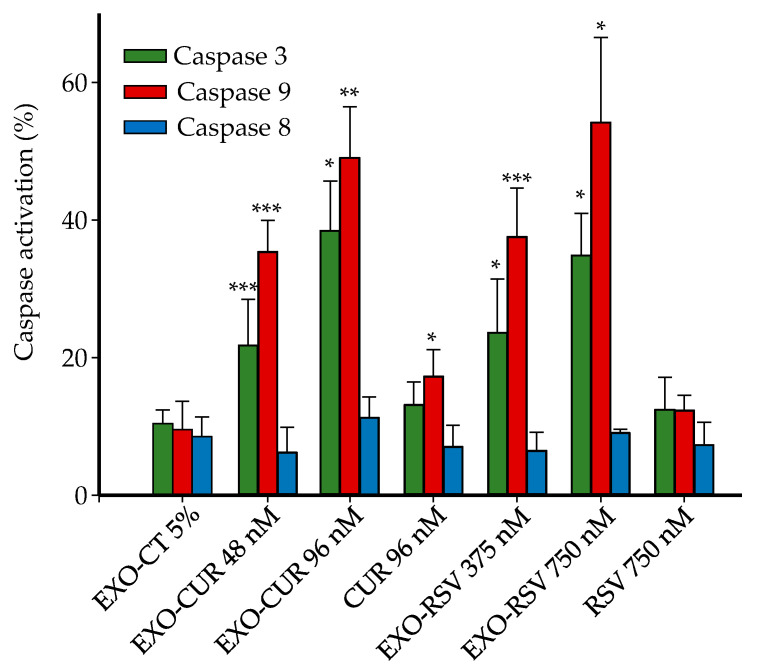
Comparison of caspases activation in MCF-7 cells exerted by CUR and RSV vs. EXO-CUR and EXO-RSV after 72 h. EXO-CT: non-loaded milk-derived EXOs. * *p* < 0.05; ** *p* < 0.01; *** *p* < 0.001 (significant differences vs. EXO-CT). Data are shown as the mean ± SD of three experiments for each treatment (n = 2 plates per experiment).

The apoptosis observed upon EXO-CUR and EXO-RSV treatments occurred via the mitochondrial (intrinsic) pathway, as indicated by the dose–response activation of caspase-9 but not caspase-8 (Figure 7). Free CUR at 96 nM showed a slight caspase-9 activation, although not enough to trigger antiproliferative activity and apoptosis under these assay conditions, as previously shown in Figure 4 and Figure 5.

### 2.5. Cellular Uptake Mechanisms of Milk-Derived Exosomes

Finally, we set out to investigate the major entry pathways in the cellular uptake of milk-derived EXOs. MCF-7 cells were co-treated for 4 h with either EXO-CUR or EXO-RSV plus specific inhibitors involved in cellular uptake mechanisms, as described in the Materials and Methods section.

**Figure 8:**
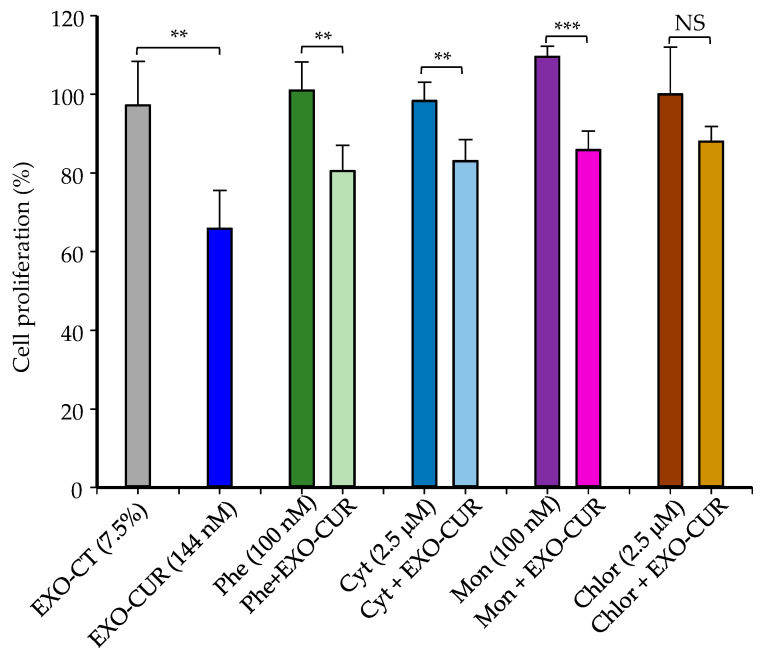
Effect of cellular uptake inhibitors on the antiproliferative activity of EXO-CUR in MCF-7 cells. Inhibitors and EXO-CUR were incubated for 4 h. Then, the cell medium was replaced by another one without inhibitors or EXO-CUR and kept for 48 h. EXO-CT: non-loaded EXOs. Phe: phenylarsine; Cyt: Cytochalasin D; Mon: monensin; Chlor: chlorpromazine. ** *p* < 0.01; *** *p* < 0.001. NS: not significantly different. Experiments were carried out three times for each treatment (n = 3 plates per experiment).

EXO-CUR at 144 nM, without cell uptake inhibitors, produced a 34% inhibition of proliferation on MCF-7 cells when measured after 48 h, but only 4 h of incubation of cells with EXO-CUR was necessary to achieve this effect (Figure 8). The antiproliferative effect of EXO-CUR was not significantly affected by phenylarsine, cytochalasin D, and monensin. However, chlorpromazine significantly reduced the antiproliferative effect of EXO-CUR under these test conditions (Figure 8).

EXO-RSV, at the highest concentration (1.1 μM), with 4 h of incubation, caused an inhibition of proliferation of 18% after evaluation at 24 h (Figure S5). In this case, EXO-RSV did not exert a significant antiproliferative effect in the presence of phenylarsine, cytochalasin D, and chlorpromazine (Figure S5).

## 3. Discussion

EXOs are communication tools that offer enormous potential for targeted bioactive compound delivery. Targeted delivery of bioactive cargoes in milk-derived EXOs has already been approached [19], especially for their potential to shuttle miRNAs and other nucleic acid molecules. However, methods for producing and purifying milk EXOs and loading them with dietary bioactives or therapeutic molecules have yet to be standardized. We have tested here three different loading methods for incorporating polyphenols into bovine-milk-derived EXOs. Overall, passive incubation was the simplest and most effective loading method.

Dietary (poly)phenols are extensively metabolized to yield phase-II conjugates with much lower activity than their unconjugated precursors [27,29,30,31]. In the present study, we first explored whether the administration of EXO-CUR and EXO-RSV could protect their cargoes (CUR and RSV) from being metabolized. In this regard, we describe here for the first time that non-conjugated, i.e., free CUR and RSV, were detected in the rat mammary tissue (41 and 300 nM, respectively) despite the low concentration (2 and 15 μM, respectively) when administered as EXO-CUR or EXO-RSV. However, no free polyphenols were detected upon administration of non-encapsulated CUR and RSV, which indicated that milk EXOs protected CUR and RSV from metabolism and delivered their cargoes in the mammary tissue. Therefore, our results suggest that the antiproliferative activity observed for EXO-CUR and EXO-RSV at the concentrations assayed in our cell models could be compatible with a plausible physiological approach [32,38].

In the present study, we could not reproduce the results of Aqil et al. [24] who encapsulated a huge CUR concentration (about 600 μM) in milk-derived EXOs and described the delivery of CUR in liver, lung, and brain rat tissues but not mammary tissues upon EXO-CUR administration. In addition, they reported exceptional results for EXO-CUR as a cancer chemopreventive agent in a cervical tumor xenograft rat model. Remarkably, these authors observed a high decrease in cell survival of 40% when assaying non-loaded EXOs as a control in MDA-MB-231 cancer cells [24]; something that we did not observe under our assay conditions.

We cannot compare our results with other reports regarding RSV encapsulation in milk-derived EXOs and their effects because this is the first study to report such encapsulation. Overall, our assay conditions for both polyphenols were challenging with regard to observing antiproliferative effects in breast cancer cells. In fact, to the best of our knowledge, we have described here for the first time the lowest CUR and RSV concentrations (assayed as EXO-CUR 96 nM and EXO-RSV 75 nM, respectively), closer to a dietary context [31,32,39] and probably compatible with their occurrence in breast tissue, showing strong antiproliferative effects on breast cancer cell models.

The antiproliferative effects were homogeneous for EXO-CUR and EXO-RSV, sharing the same mechanisms. In the present study, both encapsulated polyphenols increased the G_0_/G_1_ phase and decreased the S phase in MCF-7 cells. However, cell cycle arrest has been reported to depend on the polyphenols, their concentration, and (or) the specific cell line assayed. In the case of MCF-7 cells, 10–30 μM CUR concentrations have been reported as increasing the G_2_/M phase [32,40,41]. In the case of RSV, Su et al. [42] observed an increase in the S phase with RSV 50 μM, while Giménez-Bastida et al. [34] described an increase in the S and G_2_/M phases at 10 μM. It is of note that these results refer to the assay of much higher concentrations of non-encapsulated RSV and CUR (from 25 to 625 times more) than those tested in the present study.

EXO-CUR and EXO-RSV induced apoptosis via the mitochondrial pathway in a p53-independent mechanism since both MCF-7 (p53 wild) and MDA-MB-231 (p53 mutant) cell lines were affected almost equally. This result is interesting because other studies have observed p53-dependent effects when comparing both cell lines [34]. However, it has been described that the p21 protein, generally regulated by p53, can also be independently regulated by different molecules [43], including CUR in MDA-MB-231 cells [32]. Therefore, although we did not determine p21 in our study, we cannot rule out the possibility that EXO-CUR and EXO-RSV altered the cell cycle and promoted apoptosis by inducing p21 in a p53-independent mechanism. In general, the pro-apoptotic pathways induced by different polyphenols may differ, i.e., mitochondrial [44], extrinsic [45], or both [46,47] pathways may occur. The mitochondrial pathway was also described by Akkoç et al. [48] in MCF-7 cells but using a much higher CUR concentration (30 μM). In the case of RSV, both intrinsic and extrinsic pathways have been described in MCF-7 cells but using higher doses (10 μM) [49].

In addition to the difference in doses tested in our study compared to others, EXO-CUR and RSV entered MCF-7 cells independently of ABC transporters, which is a crucial differential feature. Our results suggest that macropinocytosis was not an essential pathway for EXO-CUR and EXO-RSV uptake. Besides, the lack of effect of the lysosome inhibitor monensin indicated that lysosomes were not involved in the intracellular transport of EXO-CUR or EXO-RSV [50]. On the contrary, EXO-CUR and EXO-RSV entered MCF-7 cells primarily via clathrin-mediated endocytosis, in agreement with other studies using milk-derived EXOs in lung cancer H1299 and colon cancer Caco-2 cells [24,51]. Previous studies have described the role of ABC transporters, especially in cancer cells, in limiting the anticancer effect of polyphenols, including CUR and RSV, by restricting their entry into the cells and (or) enhancing their efflux back to the extracellular space [34,36]. Therefore, our results suggest that EXO-CUR and EXO-RSV avoid ABC’s restriction, enter the cells by endocytosis and exert a rapid triggering of antiproliferative molecular events (4 h), evidenced after 48–72 h in cell cycle alteration and apoptosis induction in MCF-7 cells.

We are aware that our study has some limitations. For example, EXOs were isolated and unequivocally identified after detecting specific EXO-associated markers. However, we acknowledge that other subtypes of extracellular vesicles could be present in the fractions. Although the low concentrations of RSV and CUR encapsulated in milk-derived EXOs have allowed the description of potent antiproliferative effects at nanomolar concentrations for the first time, the encapsulation process could be improved to facilitate the delivery of higher polyphenol concentrations to the mammary tissue. Finally, fluorescent labeling of milk-derived EXOs could allow the assaying of higher endocytosis inhibitor concentrations to track the uptake of EXOs in real time and to more precisely differentiate the pathways involved. In the present study, our results suggest that the clathrin-mediated pathway seems to be the primary route, but we cannot unequivocally discard the possibility of cell uptake of milk EXOs following other pathways, and thus this requires further research.

## 4. Materials and Methods

### 4.1. Reagents

Resveratrol (RSV, 3,5,4′-trihydroxy-*trans*-stilbene, 99% purity), curcumin (CUR, 95%), trypan blue, bovine serum albumin (BSA), MTT (3-(4,5-dimethylthiazol-2-yl)-2,5-diphenyltetrazolium bromide), an Annexin V/PI detection kit, RNase, staurosporine, the ABC transporter inhibitors Ko143, CP100356, and probenecid, and the inhibitors of cellular uptake phenylarsine oxide (Phe), monensin (Mon), chlorpromazine (Chlor) and cytochalasin D (Cyt) were purchased from Sigma-Aldrich (St. Louis, MO, USA). FAM-FLICA caspase activation kits (3, 8, and 9) were obtained from ImmunoChemistry Technologies (Bloomington, MN, USA). Phosphate buffer saline (PBS) was obtained from Fisher Scientific (Hampton, NH, USA), and dimethylsulphoxide (DMSO), methanol (MeOH), ethanol (EtOH), and ethyl acetate (EtOAc) from Panreac (Barcelona, Spain). Ultrapure Millipore water (Bedford, MA, USA) was used throughout the study.

### 4.2. Preparation, Purification, and Detection of Milk-Derived EXOs

Raw bovine milk was collected from a local farm (Madrid, Spain). Milk-derived EXOs were isolated and purified as previously described [23], using sequential centrifugation, ultracentrifugation, and size exclusion chromatography (SEC) steps. The protein concentration of each eluted fraction after SEC was determined by the BCA method (Thermo Scientific, Waltham, MA, USA), using BSA as the standard and following the manufacturer’s instructions. The identification of EXOs was assessed by Western blot (WB) analysis using anti-Hsp90 (610418, BD, Madrid, Spain), anti-CD63 (bs-1523R, Bioss, Woburn, MA, USA), anti-TSG101 (A303-506A, Bethyl, Montgomery, TX, USA), anti-calnexin (A303-694A, Bethyl), and anti-β-casein (ab112595, Abcam, Cambridge, UK) as primary antibodies. Anti-rabbit or anti-mouse conjugated secondary antibodies were used with either Alexa Fluor^TM^ 680 or IRDye^®^ 800. Blots were visualized using an Odyssey^®^ infrared imaging system (LI-COR, Lincoln, NE, USA) and analyzed for image processing with the Image Studio Lite 5.2.5 software (LI-COR) [23].

### 4.3. CUR and RSV Loading into Milk-Derived EXOs

CUR and RSV were loaded into exosomes following three different loading methods: passive incubation, sonication, and electroporation.

#### 4.3.1. Polyphenols Preparation

CUR or RSV were dissolved in EtOH:PBS (1:1) and then exposed to exosomes (10%) at a final concentration of 250 µg/mL, reaching a 5% maximum concentration of EtOH in the EXO solution.

#### 4.3.2. EXOs Loading Methods

For passive incubation, the EXO solution was left for 1 h at 37 °C in the dark. Sonication methods consisted of (i) 4 cycles of 20 s and 2 min of waiting time in a sonicator or (ii) 6 cycles of 2 s and 2 min waiting time between cycles using a 1 Hz 150 W sonicator (J.P. Selecta, Barcelona, Spain). Electroporation was performed as previously reported [52]. Briefly, two conditions were tested: (i) 400 V, 2 pulses of 1 s and 5 s of waiting time between cycles or (ii) 400 V, 2 pulses of 0.5 msec and 5 s of waiting time between cycles. After electroporation, EXO solutions were purified by SEC to remove non-loading polyphenols. Next, EXO fractions loaded with polyphenols were pooled and concentrated by ultracentrifugation, and finally, purified milk-derived EXOs were aliquoted and stored at −80 °C for further assays.

### 4.4. Characterization of Milk-Derived EXOs

#### 4.4.1. Nanoparticle Tracking Analysis (NTA)

EXOs’ particle size distributions and concentrations were measured by NTA with a LM10 nanoparticle characterization system (NanoSight, Malvern, UK). Three replicates of 60 sec capture videos were performed using a 638 nm laser, and analysis was performed using a NanoSight NTA 3.1. program. EXO samples were appropriately diluted (1:1000–1:10,000) using 1 × PBS, and the size distributions and concentrations were analyzed at 23–27 °C. NTA analysis was performed on thawed samples.

#### 4.4.2. Transmission Electron Microscopy (TEM)

Milk-derived EXOs were visualized by TEM with negative staining. First, EXOs were placed on copper grids (200 mesh) for 3 min. Next, the samples were stained by adding 30 µL of phosphotungstic acid hydrate 1% (PTA 1%) on the grid for 1 min. Then, the excess solution was removed with filter paper, and each sample was air-dried before examining at 100 kV on a JEOL JEM 1400 TEM at the Spanish National Centre for Electron Microscopy (ICTS, Madrid, Spain).

### 4.5. Kinetic Disposition of CUR and RSV in the Rat Mammary Tissue

The Ethics Committee for Animal Experimentation (University of Murcia, Spain) and the local government (reference 624/2020) approved the study. The experimental protocol followed the Directive of the European Council 63/2010/UE and the guidelines of the Spanish government (RD 53/2013). Female Sprague–Dawley rats (230–250 g) were provided by the Experimental Animal Facility of the University of Murcia. The rats were housed 3–4 in a cage in a room with controlled temperature (22 ± 2 °C), 55 ± 10% relative humidity, and a 12 h light–dark cycle. Animals were fed a rat standard chow diet (Panlab, Barcelona, Spain). Diet and tap water were administered ad libitum until the start of the experiments.

EXO-CUR (300 μL, equivalent to 0.45 mg EXO protein, containing 2 μM CUR) or EXO-RSV (300 μL, equivalent to 0.78 mg EXO protein, and containing 15 μM RSV) were injected into the tail vein of isoflurane-sedated rats. After 1.5, 3, 6, and 15 min of exosomes administration (a minimum of n = 3 per time point), sedated rats were sacrificed using a CO_2_ chamber. In parallel, the same approach was followed for administering free CUR (2 μM) or RSV (15 μM) dissolved in PBS:EtOH (95:5).

### 4.6. Sample Processing and Polyphenol Analysis

Purified milk-derived EXOs containing CUR (200 μL, 0.3 mg/mL) or RSV (200 μL, 0.5 mg protein/mL) were mixed with ethyl acetate (1:4) acidified with 0.1% formic acid, vortexed for 1 min, and sonicated for 5 min. After centrifugation at 10,000× *g* for 5 min at 4 °C, the organic phase was collected and reduced to dryness in a speed vacuum concentrator. The evaporated samples were re-suspended in MeOH, filtered through a 0.22 µm polyvinylidene fluoride filter, and analyzed.

After the animals were sacrificed, blood samples were collected in EDTA-treated tubes by cardiac puncture and processed as described elsewhere [39]. Next, the mammary tissue was collected, extensively washed with PBS to avoid external blood contamination, and processed as previously reported [39].

CUR and derived metabolites in exosomes, blood, and mammary tissue were analyzed using UPLC-ESI-QTOF-MS as previously described [32]. RSV and derived metabolites were analyzed using GC-MS as reported elsewhere [53] (Supplementary Material).

### 4.7. Cell Lines and Assay Conditions

MCF-7 estrogen receptor (ER)-positive breast adenocarcinoma (p53 wild), MDA-MB-231 ER-negative breast adenocarcinoma (p53 mutant), and MCF-10A breast non-tumorigenic epithelial human cell lines were obtained from the American Type Culture Collection (ATCC, Manassas, VA, USA). Cells were grown according to Giménez-Bastida et al. [34].

Each cell line was exposed to CUR- or RSV-loaded EXOs (EXO-CUR and EXO-RSV, respectively) at concentrations found in the rat mammary tissue for 4–72 h, depending on the assay. In parallel, free CUR and RSV (non-encapsulated in EXOs) at similar concentrations and non-loaded EXOs (EXO-CT, controls) were also assayed.

### 4.8. Cell Viability Assays

We first determined the maximum milk-derived EXO percentage in the cell culture without affecting the osmolarity and pH of the cell, using a 5520 vapor pressure osmometer (VAPRO, Wescor, Logan, Utah, USA) and pH indicator paper (Neutralit, pH 5.5-9.0, Merck) inside the incubator. Then, the effects of free RSV (375 nM) and free CUR (48 nM) or EXO-RSV (75, 150, 300, 375, and 750 nM) and EXO-CUR (9.6, 19.2, 38, 48, and 96 nM) on MCF-7, MDA-MB-231, and MCF-10A cell viability and proliferation were measured using the MTT reduction assay according to Giménez-Bastida et al. [54]. Data are presented as the mean ± standard deviation (SD) of at least three independent experiments (n = 6 wells per experiment).

The antiproliferative effect of free CUR and RSV, and EXO-CUR and EXO-RSV on MCF-7 cells was assayed in the presence and absence of a mixture containing the three ABC inhibitors (1 μM each) after 72 h. The inhibitors were co-incubated with free CUR (48 nM) or RSV (375 nM) and the corresponding EXO-CUR and EXO-RSV at the same concentrations for 72 h.

### 4.9. Cell Cycle Analysis

The effects of free CUR (48 nM) and free RSV (375 nM) or EXO-CUR (48 nM) and EXO-RSV (375 nM) for 3 days of treatment on cell cycle distribution in both MCF-7 cell lines were measured as described previously [34]. Data are shown as the mean ± SD of 3 independent experiments (2 wells per treatment) for each time point.

### 4.10. Assessment of Apoptosis Induction

The apoptosis induction (by identifying both early and late apoptosis) exerted by free CUR (96 nM) and free RSV (375 nM) or EXO-CUR (48 and 96 nM) and EXO-RSV (375 and 750 nM) for 3 days of treatment was examined using the Annexin V/PI detection kit (Molecular Probes, ThermoFisher Scientific, Madrid, Spain) as described previously [55]. Briefly, 25,000 MCF-7 cells per sample were analyzed by flow cytometry (Coulter, EPICS XL-MCL, Miami, USA), and staurosporine 5 μM was used as a positive control. Data are shown as the mean ± SD of 3 independent experiments (2 wells per treatment) for each time point.

### 4.11. Caspase Activation Assay

The activation of caspase-3, -8, and -9 was evaluated by flow cytometry using the carboxyfluorescein (FAM) FLICA apoptosis detection kits FAM-DEVD-FMK, FAM-LETD-FMK, and FAM-LEHD-FMK, respectively (ImmunoChemistry Technologies LLC, Bloomington, MN, USA) [56]. Experiments were carried out three times for each treatment (*n* = 2 plates per experiment) in an FL1-A channel (Coulter, EPICS XL-MCL). A minimum of 2 × 10^4^ cells were analyzed for each sample.

### 4.12. Cellular Uptake of Milk Exosomes

MCF-7 cells were co-treated with either EXO-CUR or EXO-RSV plus specific inhibitors involved in cellular uptake mechanisms (phenylarsine oxide as a general endocytosis inhibitor and chlorpromazine, involved in clathrin-mediated endocytosis), microtubule assembly (cytochalasin D), and lysosome binding (monensin) [50]. After evaluating the cell viability for increasing inhibitor concentrations and incubation times (results not shown), non-cytotoxic doses of each inhibitor were selected (100 nM for phenylarsine oxide and monensin and 2.5 µM for cytochalasin D and chlorpromazine), together with a maximum incubation time of 4 h. For the proliferation analysis, MCF-7 cells were seeded in 96-well plates at a cell density of 10,000 cells/well and incubated for 24 h before adding the compounds to be evaluated. Once the incubation time had elapsed, the cells were treated for 4 h with the respective treatments, i.e., EXO-CUR (144 nM) or EXO-RSV (1.1 μM), and with each inhibitor individually. EXO-CUR and EXO-RSV concentrations corresponded to the maximum EXO percentage (7.5%) in the cell culture without affecting osmolarity in the cell medium. After incubation for 4 h, the medium was removed, and the cells were incubated for an additional 48 h with a fresh culture medium without additional treatment, i.e., without EXO-CUR, EXO-RSV, or inhibitors. After 48 h, the cellular proliferation was quantified by the MTT method, as previously described. Experiments were carried out three times for each treatment (*n* = 3 plates per experiment).

### 4.13. Statistics

Data are expressed as the mean ± SD. The empirical distribution of data with the normality assumption was tested using the Shapiro–Wilk test. The comparison of the data according to treatments, was evaluated via parametric statistics (Student’s *t*-test) or non-parametric statistics (Mann–Whitney U test) depending on whether the data presented a normal or non-normal distribution, respectively. Graphics and figures were prepared using SigmaPlot 14.5 (Systat Software, San Jose, CA, USA) and MS Office Professional Plus 2016 (Microsoft, Redmond, WA, USA). Statistically significant differences were considered at * *p* < 0.05, ** *p* < 0.01, and *** *p* < 0.001.

## 5. Conclusions

To the best of our knowledge, we have described the delivery of CUR and RSV to mammary tissue for the first time via administration of milk-derived EXO-CUR and EXO-RSV. Furthermore, this is the first report on the fast and robust antiproliferative activity, apoptotic induction, cell cycle alteration, and caspases activation exerted by nanomolar concentrations of EXO-CUR and EXO-RSV in two different human breast cancer cells, probably compatible with the CUR and RSV concentrations occurring in breast tissue, and without affecting non-tumorigenic breast cells. Remarkably, the administration of both free (non-encapsulated) polyphenols failed to exert the same effects observed for EXO-CUR and EXO-RSV at the same concentrations. Our results suggest that the internalization of EXO-CUR and EXO-RSV via clathrin-mediated endocytosis could escape the ABC transporter-mediated chemoresistance mechanisms of breast cancer cells, releasing free CUR and RSV inside the cells to exert their fast and potent anticancer effects. This study offers new scenarios for further in vivo investigation of milk-derived EXOs as nanocarriers to enhance the tissue delivery and biological effects of (poly)phenols, including CUR and RSV. Specific future actions might include validating our results in breast-cancer-induced animal models to compare free CUR and (or) RSV effects vs. those exerted by EXO-CUR and (or) EXO-RSV.

## Data Availability

The data presented in this study are available on request from the corresponding author.

## Supplementary Materials

The following supporting information can be downloaded at: https://www.mdpi.com/article/10.3390/ijms23052860/s1.
