# Supplementary material for: Milk-Derived Exosomes as Nanocarriers to Deliver Curcumin and Resveratrol in Breast Tissue and Enhance Their Anticancer Activity
Source: Int J Mol Sci. 2022 Mar 5;23(5):2860. doi: 10.3390/ijms23052860 (PMC8911159; doi:10.3390/ijms23052860)
Supplement: Supplementary file 1 [file ijms-23-02860-s001.zip › ijms-1594724-supplementary.pdf]

SUPPLEMENTARY MATERIAL

Milk-Derived Exosomes as Nanocarriers to Deliver Curcumin and Resveratrol in Breast Tissue and Enhance Their Anticancer Activity

Antonio González-Sarrías [1], Carlos E. Iglesias-Aguirre [1], Adrián Cortés-Martín [1,2], Fernando Vallejo [1], Alice Cattivelli [1,3], Lorena del Pozo-Acebo [4], Andrea Del Saz [4], María Carmen López de las Hazas [4], Alberto Dávalos [4] and Juan Carlos Espín [1,*]

[1] Laboratory of Food and Health, Research Group on Quality, Safety, and Bioactivity of Plant Foods, Department Food Science and Technology, CEBAS-CSIC, P.O. Box 164, Campus de Espinardo, 30100 Murcia, Spain; agsarrias@cebas.csic.es (A.G.-S.); ceiglesias@cebas.csic.es (C.E.I.-A.); acortesmartin@ucc.ie (A.C.-M.); fvallejo@cebas.csic.es (F.V.); alice.cattivelli@unimore.it (A.C.)
[2] APC Microbiome Ireland & School of Microbiology, University College Cork, T12 YT20 Cork, Ireland
[3] Department of Life Sciences, University of Modena and Reggio Emilia, Via Amendola 2—Pad. Besta, 42100 Reggio Emilia, Italy
[4] Laboratory of Epigenetics of Lipid Metabolism, Madrid Institute for Advanced Studies (IMDEA)-Food,
CEI UAM + CSIC, 28049 Madrid, Spain; lorena.delpozo@imdea.org (L.d.P.-A.); andrea.delsaz@imdea.org (A.D.S.); mcarmen.lopez@imdea.org (M.C.L.d.l.H.); alberto.davalos@imdea.org (A.D.)
* Correspondence: jcespin@cebas.csic.es

Supplementary Methods

UPLC-QTOF-MS analyses

Analyses were performed on an Agilent 1290 Infinity UPLC system coupled to a 6550 Accurate-Mass quadrupole-time-of-flight (QTOF) mass spectrometer (Agilent Technologies, Waldbronn, Germany) using an electrospray interface (Jet Stream Technology). Separation was achieved on a reverse-phase Poroshell 120 EC-C18 column (3 x 100 mm, 2.7 μm) (Agilent) operating at 30ºC. The mobile phases were water:formic acid (99.9:0.1 v/v) (Phase A) and acetonitrile:formic acid (99.9:0.1 v/v) (Phase B). Gradient program was as follows: 0-3 min, 5-15%B; 3-11 min, 15-30%B; 11-15 min, 30-50%B, 15-21 min, 50-90%B. Finally, the B content was decreased to the initial conditions (5%) in 1 min, and the column re-equilibrated for 5 min. The flow rate was set constant at 0.4 mL/min, and the injection volume was 3 μL. The optimal conditions of the electrospray interface were as follows: gas temperature 280ºC, drying gas 9 L/min, nebulizer 45 psi, sheath gas temperature 400ºC, sheath gas flow 12 L/min. Spectra were acquired in single MS mode with *m/z* range of 100-1100, negative polarity, and an acquisition rate of 1.5 spectra/s. Internal mass calibration by simultaneous acquisition of reference ions and mass drift compensation was used for obtaining low mass errors. Data were processed using the Mass Hunter Qualitative Analysis software (version B.06.00, Agilent). In the case of mammary tissue, a previously validated method was used [1]. The exact masses of curcumin (CUR), resveratrol (RSV), and their derived metabolites were extracted using an extraction window of 0.01 *m/z*. The identification of compounds was carried out by taking information about their elution order, molecular weight, fragmentation by MS/MS, and, chromatographic comparison with an authentic standard. CUR, RSV and derived metabolites were quantified by peak area integration of their extracted ion chromatograms

(EICs). The method was validated for linearity, precision intra- and inter-day, limits of detection (LOD), and quantification (LOQ) as well as for matrix effect as previously described [1].

GC-MS analyses

Processed mammary tissue samples [1] were dissolved in 30 µl of pyridine and converted to trimethylsilyl derivatives by adding 30 µl of N,O-bis-(trimethylsilyl) trifluoroacetamide (BSTFA) containing 1% trimethylchlorosilane (TMCS). The chemical reaction was performed by heating to 100°C for 15 min. Then, 1 µl of this reaction mixture was injected into the gas chromatograph. Silylated samples were analyzed using a HP 8890 gas chromatograph interfaced with a HP 5977B mass selective detector (MSD). A HP5-MS (30 m x 0.25 mm ID and film thickness of 0.25 µm) phase capillary columns (30 m x 0.25 mm ID, 0.25 µm film thicknesses) was used with helium as a carrier gas at a constant rate of 1 mL/min. The temperature of the injector and MS source was maintained at 200 °C. The column temperature program consisted of injection at 60°C (hold time 1 min), which was raised at 7°C/min to 180°C, at 3°C/min to 200°C (hold time 1 min), and finally at 10°C/min to 230°C (hold time 10 min). The samples were analyzed in splitless mode with a purge flow to split vent of 50mL/min at 0.8min. An Agilent 5190-2292-900µL (Splitless, single taper, ultra inert) liner was used. The MS was operated in the electron impact mode with ionization energy of 70 eV. The scan mass range was set from 100 to 800 Da at 2.0 scan/sec. The mass spectrum was acquired in positive, electron impact (70 eV), low-resolution mode. Trimethylsylil resveratrol was determined and quantified from a reconstructed ion chromatogram using *m/z* 444. Quantitation was performed from a calibration curve of a standard sample of sylilated RSV.

References

1. Ávila-Gálvez, M.Á.; González-Sarrías, A.; Martínez-Díaz, F.; Abellán, B.; Martínez-Torrano, A.J.; Fernández-López, A.J.; Giménez-Bastida, J.A.; Espín, J.C. Disposition of Dietary Polyphenols in Breast Cancer Patients' Tumors, and Their Associated Anticancer Activity: The Particular Case of Curcumin. *Mol. Nutr. Food Res.* **2021**, *65*, e2100163. https://doi.org/10.1002/mnfr.202100163.
2. Joseph, J.A.; Fisher, D.R.; Cheng, V.; Rimando, A.M.; Shukitt-Hale, B. Cellular and behavioral effects of stilbene resveratrol analogues: Implications for reducing the deleterious effects of aging. *J. Agric. Food Chem.* **2008**, *56*, 10544–10551. https://doi.org/10.1021/jf802279h.

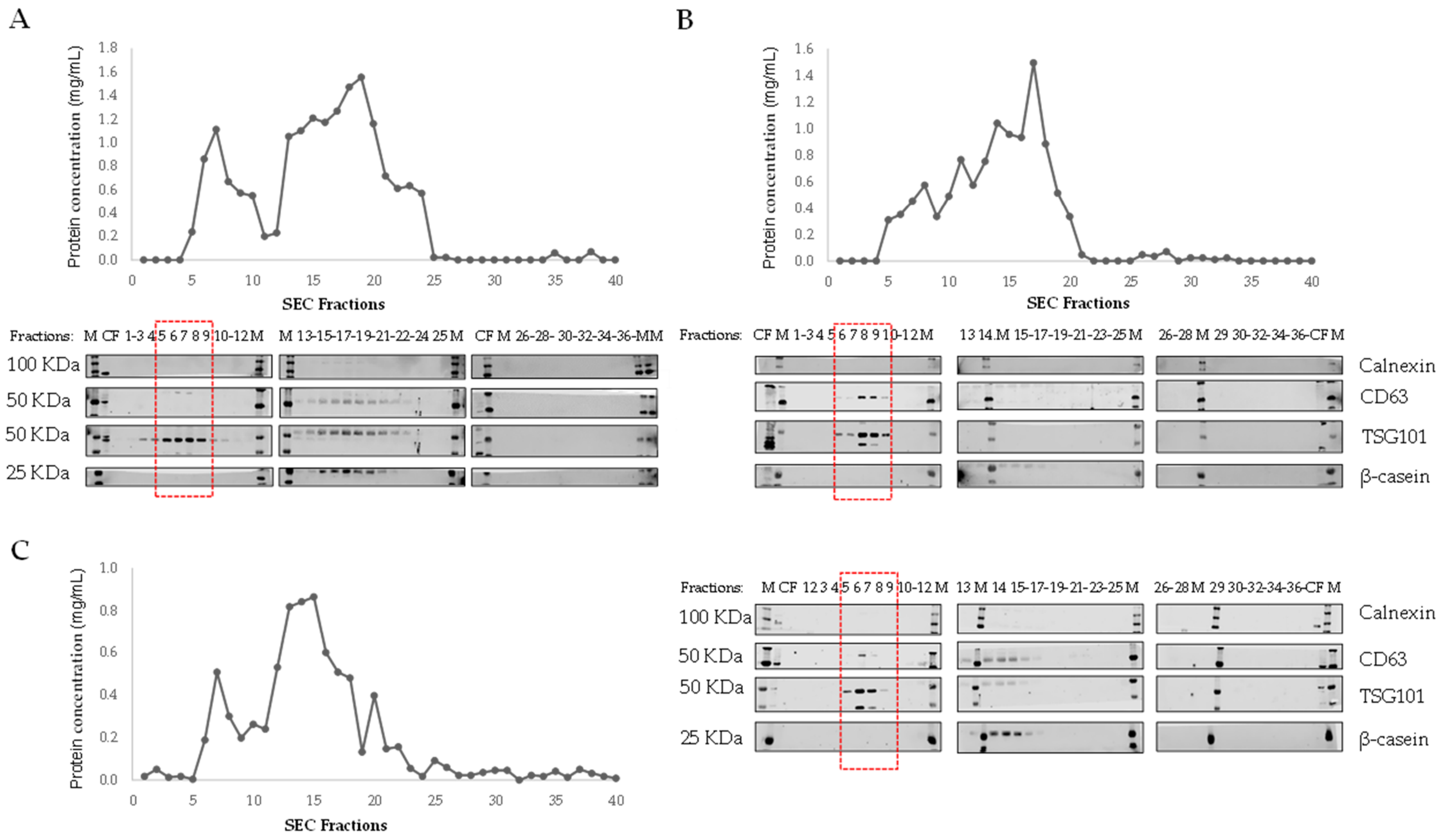
A
B
C
Protein concentration (mg/mL)
SEC Fractions
Fractions: M CF 1-3 4 5 6 7 8 9 10-12 M
M 13-15-17-19-21-22-24 25 M
CF M 26-28- 30-32-34-36-MM
100 KDa
50 KDa
50 KDa
25 KDa
Fractions: CF M 1-3 4 5 6 7 8 9 10-12 M
13 14 M 15-17-19-21-23-25 M
26-28 M 29 30-32-34-36-CF M
Calnexin
CD63
TSG101
β-casein
Fractions: M CF 1 2 3 4 5 6 7 8 9 10-12 M
13 M 14 15-17-19-21-23-25 M
26-28 M 29 30-32-34-36-CF M
100 KDa
50 KDa
50 KDa
25 KDa
Calnexin
CD63
TSG101
β-casein

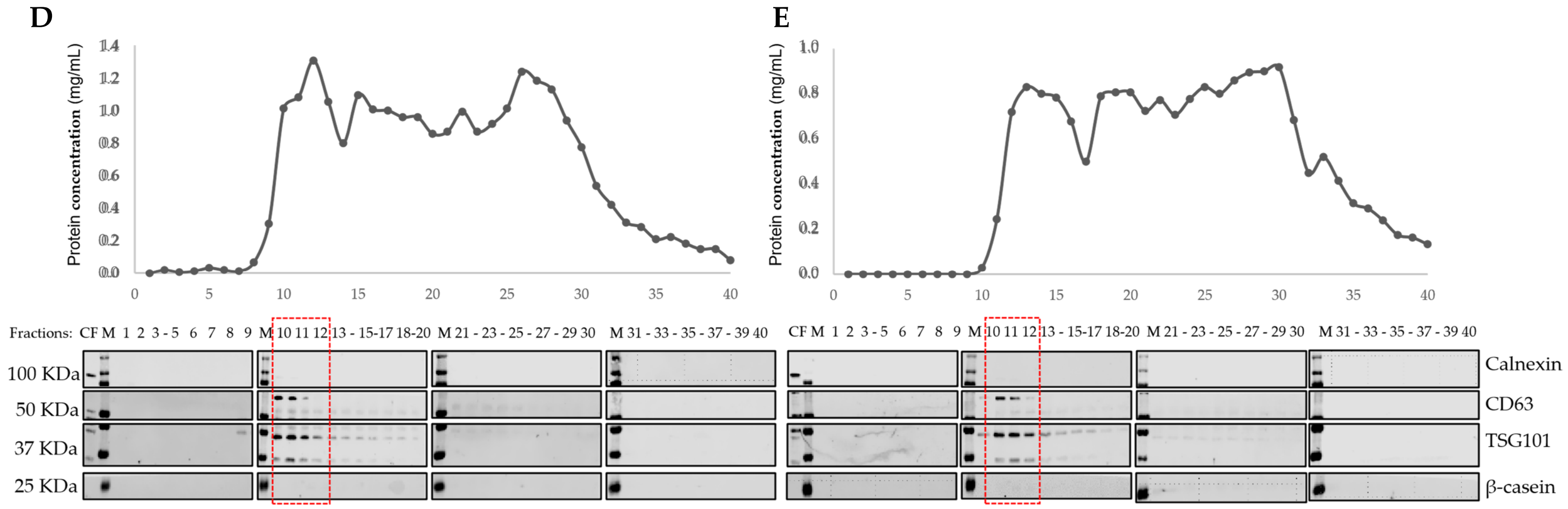

Figure S1. Identification of milk-derived EXOs by WB after sonication, passive incubation or electroporation with CUR and(or) RSV. Fractions were collected after ultracentrifugation, loading with the indicated polyphenol, and further purified by size exclusion chromatography (SEC). (**A**) Identification of EXO-CUR fractions after ultrasound loading. (**B**) Identification of EXO-RSV fractions after ultrasound loading. (**C**) Identification of RSV fractions after direct passive incubation. (**D**) Identification of EXO-CUR fractions after electroporation loading. (**E**) Identification of EXO-RSV fractions after electroporation loading. Red dashes: fractions with purified milk-derived EXOs (negative for calnexin and -casein and positive for CD63 and TSG101). CF, Human cell fraction (positive for calnexin); M, molecular weight marker; CUR, curcumin; RSV, resveratrol.

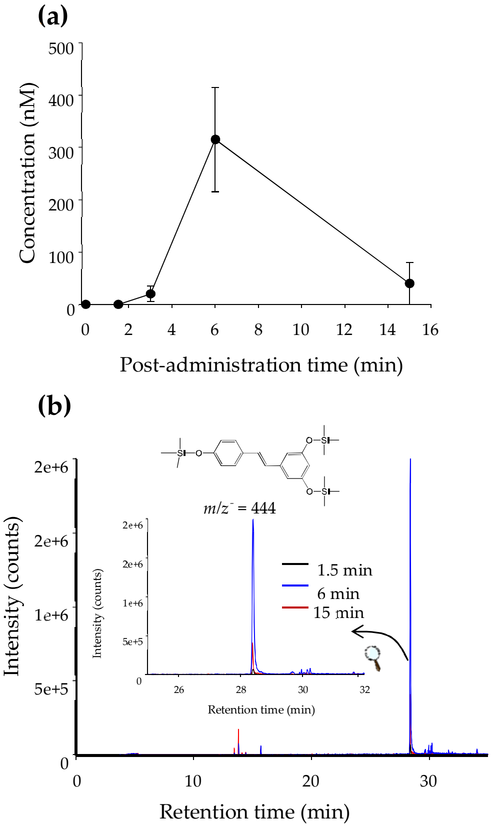

Figure S2. (**a**) RSV kinetic distribution in the rat mammary tissue. (**b**) The extracted ion chromatograms shows the molecular ion of trimethylsylil resveratrol after 1.5, 6, and 15 min of EXO-RSV intravenous administration. A minimum of 3 animals were used for each time point. Data are shown as mean±standard deviation (SD).

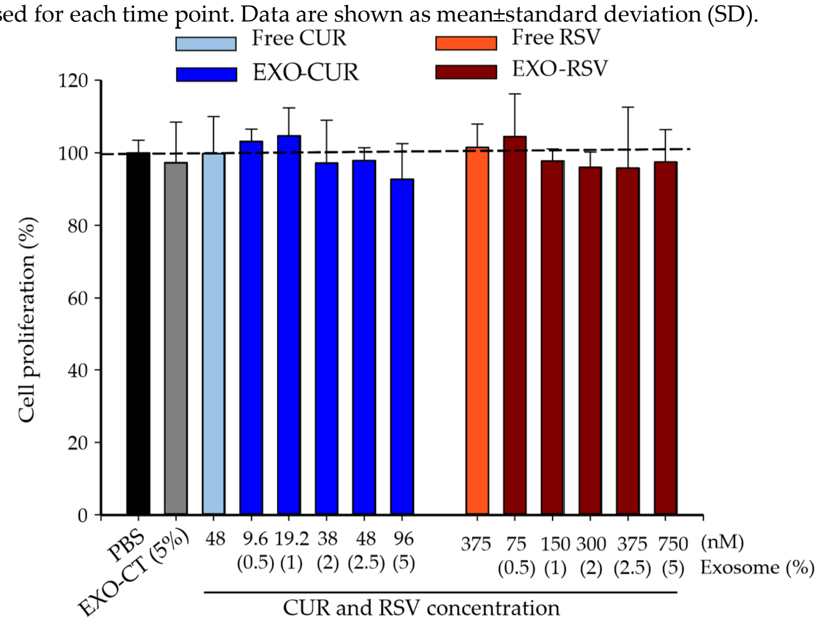

Figure S3. Comparison of the effect of free RSV and CUR *vs.* EXO-RSV and EXO-CUR on MCF-10A cells viability after 72 h. EXO-CT, control (non-loaded) EXOs. The X-axis shows the concentration of CUR and RSV incorporated by EXOs, and

below the % of the EXOs fraction tested in the cell media. No significant differences were observed for any treatment. Data are presented as the mean±SD of at least three independent experiments (n=6 wells per experiment).

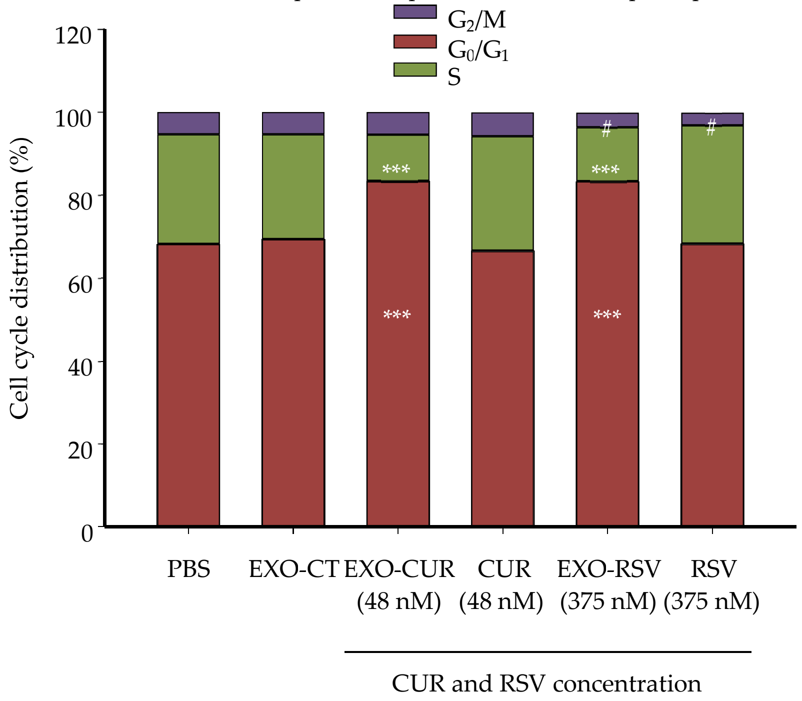

Figure S4. Comparison of the effect of RSV and CUR *vs.* EXO-RSV and EXO-CUR on the cell cycle distribution of MCF-7 cells after 72 h. ***$P < 0.001$, #$P < 0.1$ (comparison of CUR and RSV *vs.* PBS, and EXO-CUR and EXO-RSV *vs.* EXO-CT). Data are shown as the mean ± SD of 3 independent experiments (2 wells per treatment) for each time point.

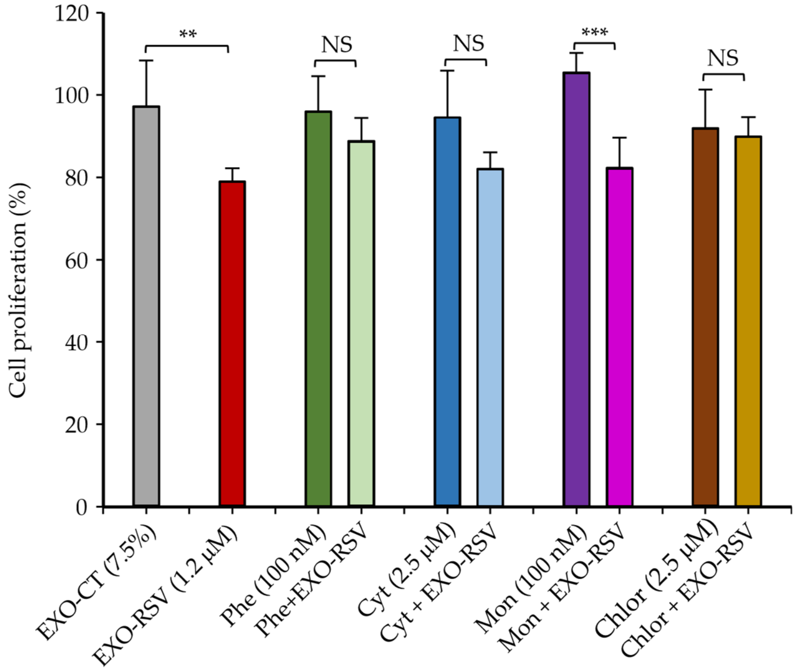

Figure S5. Effect of cellular uptake inhibitors on the antiproliferative activity of EXO-RSV in MCF-7 cells. Inhibitors and EXO-RSV were incubated for 4 h. Then, the cell medium was replaced by another one without inhibitors or EXO-RSV and kept for 48 h. EXO-CT, control (non-loaded) EXOs. Phe, phenylarsine; Cyt, Cytochalasin D; Mon, monensin; Chlor, chlorpromazine. *$p < 0.05$, **$p < 0.01$, $p < 0.001$. NS, Not significantly different. Experiments were carried out three times for each treatment (n = 3 plates per experiment).
